# Pictorial essay: The many faces of craniosynostosis

**DOI:** 10.4103/0971-3026.76055

**Published:** 2011

**Authors:** Paritosh C Khanna, Mahesh M Thapa, Ramesh S Iyer, Shashank S Prasad

**Affiliations:** 1 Department of Radiology, Seattle Children’s Hospital/University of Washington School of Medicine, Seattle, Washington, USA; 2 Center for Integrative Brain Research, Seattle Children’s Research Institute, Seattle, Washington, USA; 3 Department of Radiology, University of Texas Medical Branch Galveston, Texas, USA

**Keywords:** Craniosynostosis, craniofacial syndromes, 3-dimensional computed tomography, magnetic resonance imaging

## Abstract

Craniosynostosis is a common condition in the pediatric age group, which may either be isolated or may present as part of a craniofacial syndrome. This pictorial review illustrates the underlying mechanisms and pathophysiology of craniosynostosis, the various types of craniosynostoses, common craniofacial syndromes and the role of imaging in their diagnosis and management.

## Introduction

Craniosynostosis, or craniostenosis, is the premature fusion of cranial sutures and may be isolated or may present as part of a craniofacial syndrome. It typically alters the shape of the cranial vault. Broad categories include “simple craniosynostosis,” involving only one suture, or “compound craniosynostosis,” where two or more sutures are involved.[1] Based on etiology, craniosynostosis may be characterized as primary (intrinsic defect in suture) or secondary (premature closure of normal sutures because of another medical condition such as deficient brain growth).[2]

## Types of Craniosynostoses and their Etiology

From an etiologic standpoint, primary craniosynostosis can be idiopathic or familial. The familial form usually manifests as a component of the various craniofacial syndromes and may result from one of several genetic mutations. The mutations that have been well characterized are mutations in fibroblast growth factor receptor-1 (FGFR1), FGFR2, FGFR3, twist homolog 1 (TWIST1) and msh homeobox 2 (MSX2) genes.[2]

Fibroblast growth factors modulate cell proliferation, differentiation and migration.[3] These growth factors act through FGFRs. Mutations in FGFR-1 through FGFR-3 have been associated with Pfeiffer, Apert, Crouzon, Beare-Stevenson, Jackson-Weiss and Muenke syndromes. Mutation in the TWIST1 gene is associated with Saethre-Chotzen Syndrome. MSX2 gene mutation is associated with the Boston-type craniosynostosis.[4]

Secondary craniosynostosis results from a known underlying disorder. This includes systemic and metabolic conditions such as hyperthyroidism, hypercalcemia, hypophosphatasia, vitamin D deficiency, renal osteodystrophy, Hurler’s Syndrome, sickle cell disease and thalassemia. Craniosynostosis can also be seen secondary to conditions that diminish growth stretch at sutures, such as microcephaly, encephalocele and shunted hydrocephalus.[5]

## Pathophysiology

Normally, calvarial bones grow perpendicular to suture lines. Premature sutural fusion begets an abnormal growth pattern, resulting in calvarial deformity. The nature of the deformity depends on which sutures are involved, the time of onset and the sequence in which individual sutures fuse. Early release of all fused sutures is necessary to restore growth.[6]

## Epidemiology

The birth prevalence of craniosynostosis ranges from 3.1 to 4.8 per 10,000 live births.[7–9] The isolated variety constitutes 80-90% of cases and the sutures most commonly involved are the sagittal, coronal, metopic and lambdoid, in descending order of frequency. The syndromic variety accounts for up to 10-20% of cases. Coronal synostosis is more frequently seen in females, while sagittal synostosis is more common in males.[1,8–10] Most cases are diagnosed early in life. Increased intracranial pressure (ICP), mental retardation, visual defects and cosmetic deformity are frequent causes of morbidity.[11–13]

## Pertinent Cranial Embryology and Anatomy

### Embryology

The skull develops from the viscerocranium (responsible for development of the facial bones) and the neurocranium (the portion of the skull that surrounds the brain). The neurocranium has cartilaginous (chondrocranium that develops from endochondral ossification and gives rise to the skull base) and membranous (dermatocranium that develops from membranous ossification and gives rise to the calvarial vault) components. The brain grows rapidly in the early years of life, with growth of the neurocranium essentially ceasing at about 7 years of age.

The fontanels usually close by the second year of life - the anterior fontanel closes by about 20 months and the posterior fontanel by about 3 months. Complete sutural fusion occurs after the third decade of life.[10,14,15]

### Anatomy

**Figure 1(A-E):**
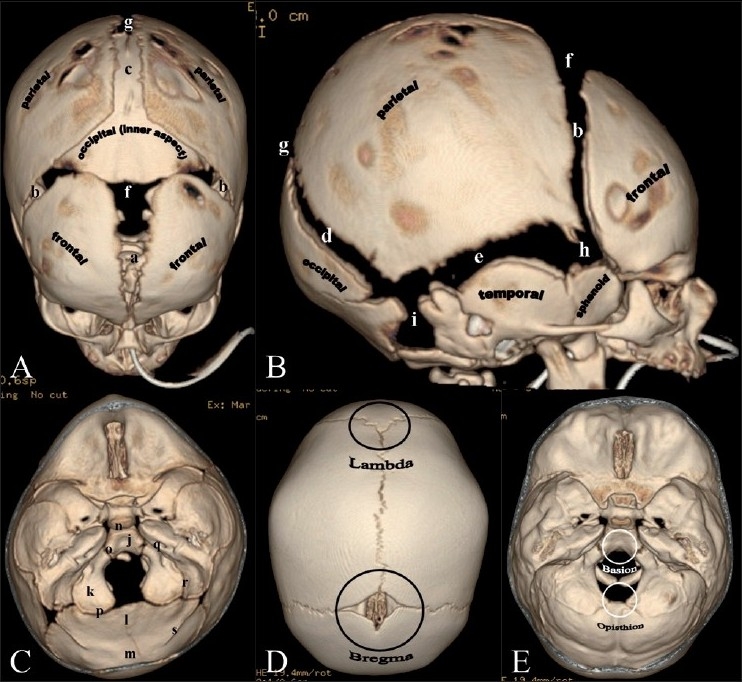
Normal sutures. 3DCT volume rendered images. Vertex (A) and lateral (B) views. (a) Metopic suture; (b) coronal sutures; (c) sagittal suture; (d) lambdoid suture; (e) squamosal suture; (f) anterior fontanel; (g) posterior fontanel; (h) sphenoidal fontanel; (i) mastoid fontanel. Cranial vault bones usually ossify from the center to periphery, which results in this “widened” appearance of the sutures in the newborn. Endocranial skull base view (C) shows portions of the occipital bone and sutures: (j) Basioccipital; (k) paired exoccipital; (l) supraoccipital; and (m) interparietal. Associated synchondroses are (n) spheno-occipital; (o)anterior intra-occipital; (p) posterior intra-occipital; (q) petro-occipital; (r) occipitomastoid; (s) and mendosal sutures. Note that o, k, p and s are paired structures. Vertex view (D) shows the lambda (point of intersection of the sagittal and lambdoid sutures) and bregma (point of intersection of the coronal and sagittal sutures. Endocranial skull base view (E) shows the basion (located on the basiocciput, at the midpoint of the anterior margin of the foramen magnum) and opisthion (located on the occipital bone, at the midpoint of the posterior margin of the foramen magnum).

Pertinent anatomy includes the major and minor sutures, fontanels, bones and major skull landmarks, all of which have been depicted in Figure 1.

## Imaging Modalities

The mainstay of craniosynostosis imaging is CT scan with 3D surface-rendered reconstructions[16] [Figure 1], including endocranial skull base views [Figure 1C]. CT scans involve administering radiation. However, at our institution, we are compliant both with the ALARA (as low as reasonably achievable) concept and the “Image Gently” recommendations (www.imagegently.org). Studies with low-dose CT head and face are obtained using a 64-slice multidetector CT scanner (GE LightSpeed VCT, Waukesha WI). Imaging parameters used are 120 kV and 150 mA (or lower depending on patient age), delivering a total dose (Volume Computed Tomography Dose Index or CTDI_vol_) of 31.26 mGy or lower. Evaluation of a half-dose protocol is currently under way at our institution (120 kV, 75 mA, CTDI_vol_ of 31.26 mGy or lower). Compared with the higher adult doses as per the American College of Radiology (ACR) CT accreditation recommendations (CTDI_vol_ of 75 mGy), we have demonstrated no substantial loss in quality of bone imaging with our pediatric CT protocols.

Rapid scanning with 64-slice multidetector scanners minimizes image degradation from patient movement; in select cases, however, patients may have to be sedated. 3D-CT allows for exquisite assessment of the vault and skull base in addition to assessment of secondary changes of craniosynostosis in the cranial fossae, orbits and facial bones.[16] Planar bone algorithm images in the axial, sagittal and coronal planes facilitate problem solving if the presence of suture fusion is unclear from the 3D images. CT scan is particularly useful in the evaluation of preoperative calvarial deformity, immediate postoperative acute intracranial abnormality (bleeds, etc. on brain algorithm/windows) and long-term postoperative follow-up of calvarial deformities.

### Other imaging modalities

USG is of limited value in the assessment of craniosynostosis. Severe cranial malformations can be recognized on antenatal USG. Postnatal use of USG in the assessment of craniosynostosis is not established.[10] Initial evaluation of certain patients may start with plain radiographs for skull shape. Normal sutures are lucent, serrated and nonlinear. Synostotic sutures are straight, sclerotic and heaped up, or absent.[17] In the majority of cases, however, sutural evaluation is inadequate with radiographs alone.

MRI affords exquisite intracranial soft tissue detail and is typically used for associated congenital anomalies. MRI is more useful than CT scan for assessment of associated anomalies and facilitates diagnosis and classification in many cases.[18]

## Types [Table 1]

### Major suture synostoses

#### Dolichocephaly and scaphocephaly [Figure 2]

**Figure 2(A-C):**
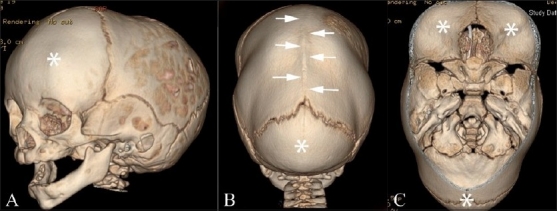
Dolichocephaly and scaphocephaly. 3DCT volume rendered images. Left anterior (A), posterior (B) and endocranial (C) images show fusion and prominent ridging of the sagittal suture (white arrows) with frontal and occipital bossing (FNx01). Also note the increased anterior-posterior dimension and restricted biparietal diameter.

Dolichocephaly (Greek: dolikhos = long) and scaphocephaly (Greek: scaphe = boat) are cranial vault deformities that result from premature fusion of the sagittal suture, with a resultant increase in the anterior-posterior dimension as well as restriction of biparietal growth. There is a male predilection.[10] Scaphocephaly forms a distinct subset of dolichocephaly, in that there is obvious ridging of the fused sagittal suture, akin to the keel of a boat. Both groups have bitemporal narrowing and may have frontal and/or occipital bossing in pronounced cases. Neurologic deficits and elevated ICP are rare.[14]

**Table 1:** Types of craniosynostoses (see text for details)

Deformity	Suture (incidence, %)
Dolichocephaly	Sagittal (50-58)
Scaphocephaly	Sagittal
Brachycephaly	Bicoronal (20-29)
Anterior plagiocephaly	Unicoronal
Turricephaly	Bilateral lambdoid (2-4)
Posterior plagiocephaly	Unilateral lambdoid
Trigonocephaly	Metopic (4-10)
Oxycephaly	Sagittal + coronal
Kleeblattschädel	Sagittal + coronal + lambdoid

(See text for details)

#### Brachycephaly and anterior plagiocephaly [Figures 3 and 4]

**Figure 3(A-E):**
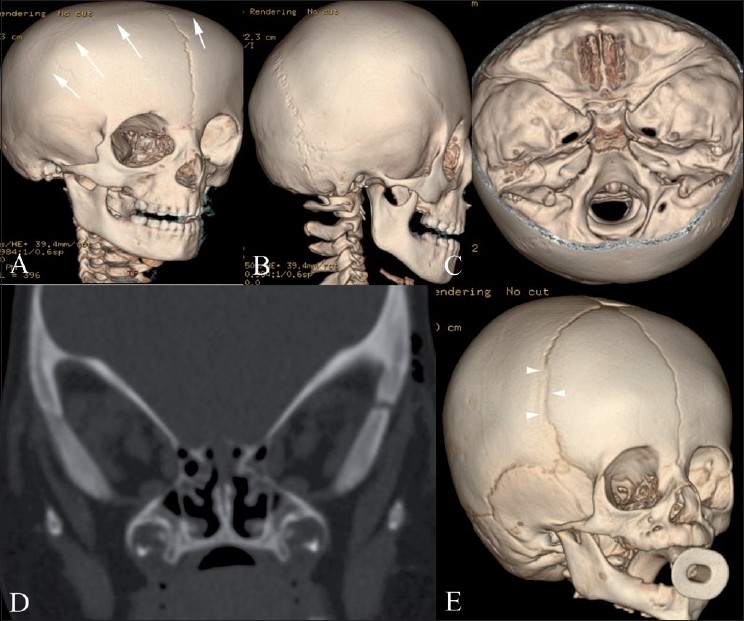
Bilateral (A-D) and unilateral partial (E) coronal synostosis 3DCT volume rendered images (A-C, E) and coronal CT scan (D). There is complete fusion of the coronal suture (white arrows) with a prominent frontal bone and flattened occiput. Coronal reconstruction (D) demonstrates prominent bilateral elliptical orbits, known as the “harlequin eye” deformity. Note the early partial fusion of the right coronal suture (arrowheads in E)

**Figure 4(A-C):**
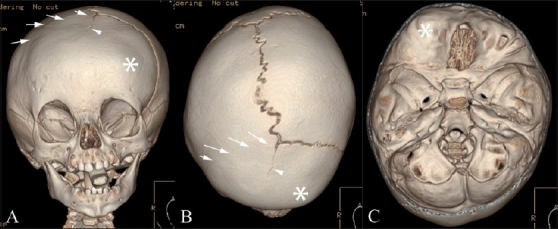
Anterior plagiocephaly. Anterior (A), vertex (B) and endocranial (C) 3DCT volume rendered images show right unicoronal synostosis (white arrows) with ipsilateral frontal flattening and contralateral frontal prominence (FNx01). An incompletely fused metopic suture is also seen (arrowhead), probably compensatory

Brachycephaly (Greek: brakhu = short) and anterior plagiocephaly (Greek: plagios = oblique) result from premature bicoronal or unicoronal fusion, respectively, with consequent restriction of anterior-posterior calvarial growth and relatively unimpeded biparietal growth. There is a female predilection.[14] The appearance should be distinguished from that of nonsynostotic or deformational type of plagiocephaly caused by *in utero* flattening which is managed without surgery.[1,19]

Bilateral coronal synostosis [Figure 3] results in a prominent frontal bone, flattened occiput and anterior displacement of the vertex. Secondary facial abnormalities include upper and midface hypoplasia and oblong, elliptical orbits (“Harlequin Eye”, Figure 3D) due to underdeveloped supraorbital ridges.

Unicoronal synostosis [Figure 4] resulting in anterior plagiocephaly manifests as flattening of the frontal bone on the affected side, with a prominent frontal eminence on the contralateral side. There may be an ipsilateral harlequin eye deformity in pronounced cases.[1]

#### Turricephaly and posterior plagiocephaly [Figures 5 and 6]

**Figure 5(A,B):**
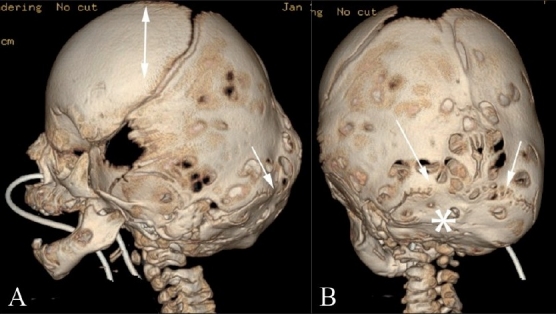
Turricephaly. Lateral (A) and posterior (B) 3DCT volume rendered images show turricephaly secondary to bilateral lambdoid fusion (arrows). Note the small, underdeveloped posterior fossa (*), and the “tall” cranium (double-headed arrow)

**Figure 6(A-C):**
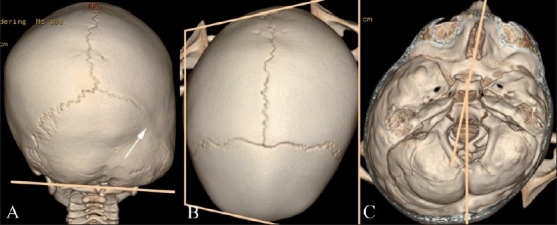
Posterior plagiocephaly. Posterior (A), superior (B) and endocranial (C) 3DCT volume rendered images show posterior plagiocephaly secondary to unilateral lambdoid fusion (arrow). The posterior skull base is tilted downward on the affected side (A), and the skull assumes a trapezoid shape (B). Note also that the posterior skull base axis (passing through the basion and opisthion) is rotated toward the side of lambdoid fusion and does not coincide with the anterior skull base axis (passing through the crista galli and basion) (C)

Lambdoid synostosis is rare. It can be unilateral or, rarely, bilateral. Bilateral lambdoid synostosis is usually found in association with syndromes. Turricephaly (Greek: turri = tower) and unilateral lambdoid synostosis are characterized by posterior plagiocephaly and represent cranial deformities secondary to bilateral and unilateral lambdoid fusion, respectively. These deformities are less common than sagittal and coronal synostoses. With bilateral lambdoid synostosis, there is flattening of the lambda and underdevelopment of the posterior fossa with unimpeded calvarial growth at the bregma, resulting in a “tall” cranium.[1] Posterior plagiocephaly [Figure 6] results in ipsilateral occipitoparietal flattening with contralateral occipitoparietal and frontal bossing.[10] The pinna on the affected side is displaced posteroinferiorly.[12] The shape of the skull as viewed from above conforms to a trapezium, and the posterior skull base is tilted downward on the side of the lambdoid fusion.[10] Also, the posterior skull base axis (passing through the basion and opisthion) is rotated toward the side of the lambdoid fusion and does not coincide with the anterior skull base axis (coursing through the crista galli and basion).

#### Deformational or positional plagiocephaly [Figure 7]

**Figure 7(A-C):**
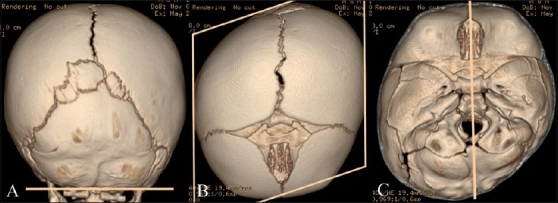
Deformational plagiocepahly. Posterior (A), superior (B) and endocranial (C) 3DCT volume rendered images. There is no identifiable synostosis. The skull shape resembles a parallelogram (B), and the posterior skull base is not abnormally tilted (A). In addition, the posterior skull base axis coincides with the anterior skull base axis (C)

Deformational or positional plagiocephaly is not a true synostosis. It is caused by compressional forces *in utero* modulated by postnatal preferential head positioning in infants sleeping on their back.[20] No synostosis is identified in these cases.

Distinction of deformational plagiocephaly from unilateral lambdoid synostosis is important as deformational/positional plagiocephaly is almost never treated surgically.[12] In positional plagiocephaly, in addition to a nonfused lambdoid suture, CT scan features include [Figure 7] ipsilateral parieto-occipital flattening, contralateral occipital bossing and ipsilateral frontal bossing. The shape of the skull as viewed from above conforms to a parallelogram, and the posterior skull base is not abnormally tilted on the side of the parieto-occipital flattening. The ipsilateral pinna on the affected side is displaced anteriorly in deformational posterior plagiocephaly.[21]

#### Trigonocephaly [Figure 8]

**Figure 8(A-C):**
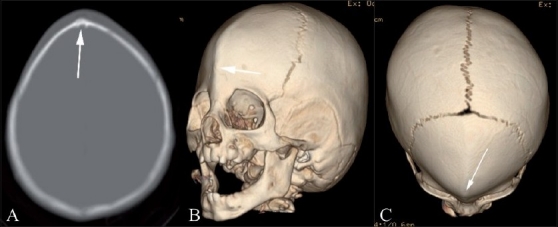
Trigonocephaly. Axial CT scan (A), anterolateral (B) and superior (C) 3DCT volume rendered images show trigonocephaly from premature / abnormal fusion of the metopic suture (arrow). Note marked flattening of the frontal bones

Trigonocephaly (Greek: trigonos = three angles) results from premature fusion of the metopic suture before 6 months (3-9 months) of age.[10] This can occur from *in utero* fusion, and one-third of the cases are syndromic and may be associated with other midline anomalies[10] involving the brain or palate. Colobomas and urinary tract abnormalities may also occur. Experienced clinicians can make this diagnosis easily, which can be confirmed with radiography or CT.[14]

The imaging hallmark of metopic craniosynostosis is trigonocephaly - a triangular, pointed forehead with flattened frontal bones and bossing of the parieto-occipital regions. Other features that can be seen are hypotelorism,[1] narrow anterior cranial fossa, hypoplasia of the ethmoid sinuses and a “quizzical” orbit appearance due to upward deviation of the medial orbital rim,[22] deficient supraorbital ridges, elongation of the inferior angle of the anterior fontanel when unfused (“frontal notching”)and tight anterior extraaxial/subarachnoid spaces.

In recent years, milder forms of this condition, termed “metopic ridge,” have been recognized and are usually not treated surgically.[1]

#### Oxycephaly [Figure 9]

**Figure 9(A, B):**
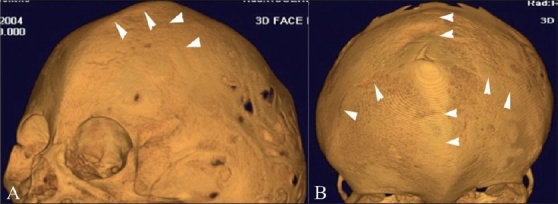
Oxycephaly. Anterolateral (A) and anterosuperior (B) 3DCT volume rendered images show oxycephaly from severe sagittal and coronal synostoses (arrowheads)

Oxycephaly (Greek: oxys = sharp) results most commonly from a combination of severe sagittal and coronal synostoses. This condition may result in microcephaly with raised ICP and neurologic impairment.[14]

#### Kleeblattschädel [Figure 10]

**Figure 10(A,B):**
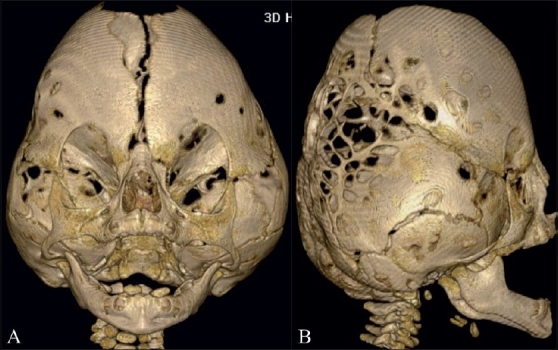
Kleeblattschädel. Anterior (A) and lateral (B) 3DCT volume rendered images show a Kleeblattschädel (cloverleaf) deformity from partial fusion of the sagittal, coronal and lambdoid sutures

Kleeblattschädel (German: kleeblatt = cloverleaf; schädel = skull, cranium) is a consequence of combined sagittal, coronal, lambdoid synostoses. The cloverleaf skull is associated with bulging temporal regions and proptotic eyes. Patients generally have severe neurological impairment.[10,18]

## Minor Sutural Synostoses [Figure 11]

**Figure 11(A, B):**
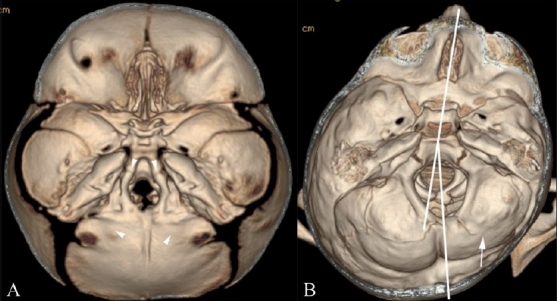
Minor suture synostoses. Endocranial 3DCT volume rendered images show symmetric (A) and asymmetric (B) minor sutural synostoses. There is symmetric fusion of the anterior and posterior intra-occipital synchondroses (arrowheads in A), associated with a small foramen magnum. Note also asymmetric fusion of the right posterior intra-occipital synchondroses (arrow in B) resulting in rotation of the posterior skull base axis towards the side of the abnormally fused suture

Minor sutures include the squamosal sutures and the synchondroses of the mid and posterior skull base [Figure 1C]: paired sphenooccipital, anterior intraoccipital, posterior intraoccipital, petrooccipital and occipitomastoid. Mendosal sutures lie at the junction of the membranous and endocranial portions of the occipital bone and normally disappear between the ages of 2 and 6 months. Abnormal fusion of the minor sutures may be symmetric [Figure 11A] or asymmetric [Figure 11B]. In the latter situation, calvarial deformity may result. The intraoccipital synchondroses may undergo asymmetric fusion [Figure 11B] with resultant tilt of the posterior skull base and rotation of the posterior skull base axis. In other instances, minor sutures, such as the mendosal, may undergo changes to compensate for major suture synostosis.[23]

## Craniofacial Syndromes

Crouzon’s, Apert’s and Pfeiffer’s syndromes are the most common craniofacial syndromes, accounting for nearly two-thirds of the syndromic cases. Most of these patients tend to exhibit raised ICP, hydrocephalus, optic atrophy and respiratory, speech and hearing problems. Surgical treatment may be necessary for cosmetic and neurologic reasons.

Noncraniofacial features include hand and foot digital anomalies (Apert’s, Pfeiffer’s), cervical fusion anomalies (Apert’s), short humerus and femur (Crouzon’s); especially important is that syndactyly is an important feature of Apert’s (acrocephalosyndactyly) and Pfeiffer’s syndromes and is very rare with Crouzon’s syndrome (“Type II” Crouzon’s may have partial syndactyly).

### Crouzon’s syndrome [Figure 12]

**Figure 12(A-C):**
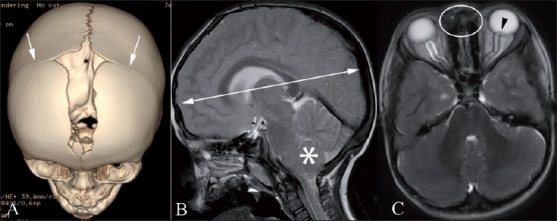
Crouzon’s syndrome. Anterosuperior 3DCT volume rendered image (A), sagittal T2W MRI (B) and axial T2W MRI (C) show premature fusion of the coronal sutures (arrows) and brachycephaly (double-headed arrow). Note also the crowded posterior fossa with onsillar herniation through the foramen magnum (FNx01). There is also hypertelorism (oval). Note the cerebrospinal fluid (CSF)-distended optic nerve sheaths bilaterally and bulging left optic disc (arrowhead) indicating raised intracranial pressure

Craniofacial features include closure of the coronal and minor skull base sutures, with brachycephaly. The overall calvarial shape depends on the order of fusion. The patients tend to be proptotic, with maxillary hypoplasia, parrot-beak nose and ocular hypertelorism. Hydrocephalus is frequently observed and there may be chronic tonsillar herniation, best depicted on MRI.[13,24]

### Apert’s syndrome [Figure 13]

**Figure 13(A-D):**
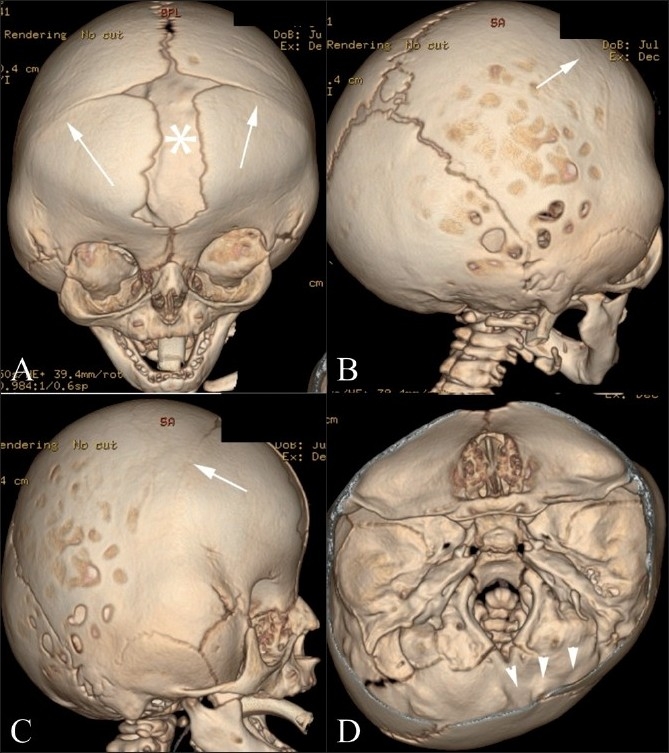
Apert’s syndrome. Frontal (A), lateral (B), right posterolateral (C) and endocranial (D) 3DCT volume rendered images show coronal synostosis (arrows), gaping frontal midline defect (FNx01), and a small malformed skull base (arrowheads)

Craniofacial features include coronal synostosis with a small, malformed skull base, large fontanels and a gaping frontal midline defect. There may be visual impairment, with an increased risk of developmental delay and mental retardation. Brain malformations, including corpus callosum agenesis, cortical atrophy or hydrocephalus, are more common with Apert’s syndrome than with Crouzon’s syndrome. There are often concomitant cervical vertebral fusion anomalies, most commonly noted at C5-C6.[2,4]

### Pfeiffer’s syndrome [Figure 14]

**Figure 14(A-C):**
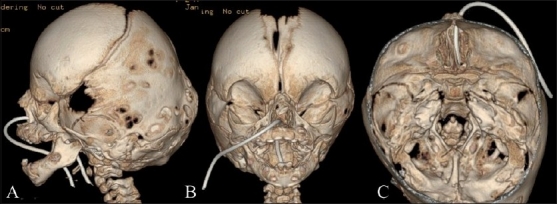
Pfeiffer’s syndrome. Lateral (A), frontal (B) and endocranial (C) 3DCT volume rendered images show a mild cloverleaf deformity and turricephaly (from bilateral partial lambdoid fusion)

There are three types of this syndrome, viz., types I, II and III. Craniofacial features of these types include the following:

Type I: Most common, hearing loss with auditory stenosis or atresia with hypoplasia or enlargement of the middle ear cavity. Patients are hyperteloric.
Type II: Cloverleaf skull, severe proptosis.
Type III: Features of types I and II with mental retardation and hydrocephalus.[25]

## Role of Imaging in Management

Conservative management is the mainstay for secondary craniosynostosis, where there is primary failure of brain growth, microcephaly and normal ICP. Conservative management is also used for positional plagiocephaly, with recommendations for changing sleep positions and use of a cranial band or helmet in more pronounced cases.[1] Follow-up evaluation with imaging is less frequently used in these cases.

**Figure 15:**
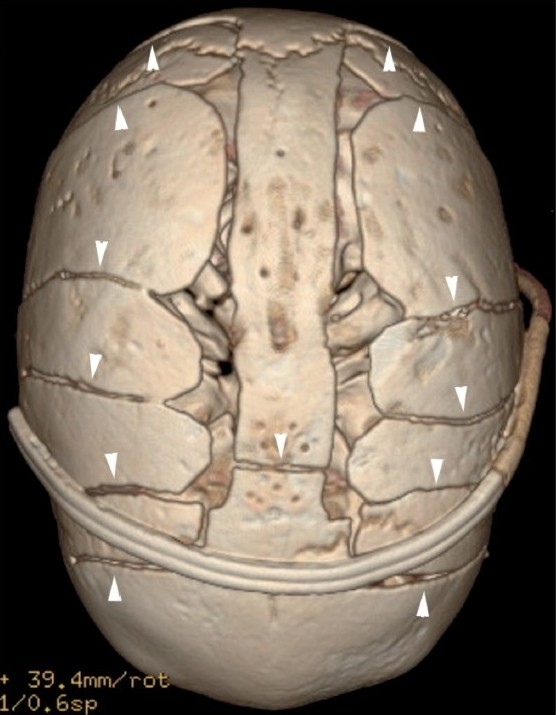
“Barrel-stave” osteotomies. Vertex 3DCT volume rendered image shows multiple parallel osteotomies (arrowheads), resembling the stave joints of a barrel. A surgical drain is present in the anterior scalp

Surgical management is typical for primary craniosynostosis, where there is obvious restriction of brain growth and raised ICP. Surgery is most effective in the first year of life. Surgery is advised as soon as the infant is able to tolerate it, usually between the ages of 3 and 9 months,[1] because the calvarial bones are malleable and heal effectively. Common surgical techniques include “strip craniectomy,” which involves the selective removal of the fused suture, “barrel-stave osteotomies” [Figure 15], which involve expansion of the vault by creating multiple parallel osteotomies, a host of other reconstructive and advancement techniques and endoscopic surgery.[10] Imaging with CT scan in the immediate postoperative period is critical to exclude acute intracranial/parenchymal events, and later in the postoperative course, for the evaluation of brain growth and cosmetic outcomes of surgery.

## References

[1] Ocal E, Sun PP, Persing JA, Albright AL, Pollack IF, Adelson PD (2007). Craniosynostosis. Principle and practice of pediatric neurosurgery.

[2] Kimonis V, Gold JA, Hoffman TL, Panchal J, Boyadjiev SA (2007). Genetics of craniosynostosis. Semin Pediatr Neurol.

[3] Wilkie AO, Patey SJ, Kan SH, van den Ouweland AM, Hamel BC (2002). FGFs, their receptors, and human limb malformations: clinical and molecular correlations. Am J Med Genetics.

[4] Jabs EW (1998). Toward understanding the pathogenesis of craniosynostosis through clinical and molecular correlates. Clin Genet.

[5] Cohen MM, Cohen MM, MacLean RE (2000). Craniosynostosis: diagnosis, evaluation and management.

[6] Kreiborg S, Cohen MM, MacLean RE (2000). Postnatal growth and development of the craniofacial complex in premature craniosynostosis. Craniosynostosis: diagnosis, evaluation and management.

[7] French LR, Jackson IT, Melton LJ (1990). A population-based study of craniosynostosis. J Clin Epidemiol.

[8] Lajeunie E, Le Merrer M, Bonaïti-Pellie C, Marchac D, Renier D (1995). Genetic study of nonsyndromic coronal craniosynostosis. Am J Med Genet.

[9] Boulet SL, Rasmussen SA, Honein MA (2008). A population-based study of craniosynostosis in metropolitan Atlanta, 1989-2003. Am J Med Genet.

[10] Kirmi O, Lo SJ, Johnson D, Anslow P (2009). Craniosynostosis: a radiological and surgical perspective. Semin Ultrasound CT MR.

[11] Aviv RI, Rodger E, Hall CM (2002). Craniosynostosis. Clin Radiol.

[12] Kotrikova B, Krempien R, Freier K, Mühling J (2007). Diagnostic imaging in the management of craniosynostoses. Eur Radiol.

[13] Kabbani H, Raghuveer TS (2004). Craniosynostosis. Am Fam Physician.

[14] Ridgway EB, Weiner HL (2004). Skull deformities. Pediatr Clin North Am.

[15] Lemire R, Cohen MM, MacLean RE (2000). Embryology of the skull. Craniosynostosis: diagnosis, evaluation and management.

[16] Vannier MW, Hildebolt CF, Marsh JL, Pilgram TK, McAlister WH, Shackelford GD (1989). Craniosynostosis: diagnostic value of three-dimensional CT reconstruction. Radiology.

[17] Benson ML, Oliverio PJ, Yue NC, Zinreich SJ (1996). Primary craniosynostosis: imaging features. AJR Am J Roentgenol.

[18] Vannier MW, Cohen MM, MacLean RE (2000). Radiologic evaluation of craniosynostosis. Craniosynostosis: diagnosis, evaluation and management.

[19] Bridges SJ, Chambers TL, Pople IK (2002). Plagiocephaly and head binding. Arch Dis Child.

[20] Mulliken JB, Vander Woude DL, Hansen M, LaBrie RA, Scott RM (1999). Analysis of posterior plagiocephaly: deformational versus synostotic. Plast Reconstr Surg.

[21] Cohen MM, MacLean RE, Cohen MM, MacLean RE (2000). Anatomic, genetic, nosologic, diagnostic, and psychosocial considerations. Craniosynostosis: diagnosis, evaluation and management.

[22] Blaser S (2008). Abnormal skull shape. Pediatr Radiol.

[23] Sze RW, Hopper RA, Ghioni V, Gruss JS, Ellenbogen RG, King D (2005). mdct diagnosis of the child with posterior plagiocephaly. AJR Am J Roentgenol.

[24] Carinci F, Pezzetti F, Locci P, Becchetti E, Carls F, Avantaggiato A (2005). Apert and Crouzon syndromes: clinical findings, genes and extracellular matrix. J Craniofac Surg.

[25] Cohen MM, Cohen MM, MacLean RE (2000). Pfeiffer Syndrome. Craniosynostosis: Diagnosis, evaluation and management.

